# Supplementary material for: Characterizing Circulating microRNA Signatures of Type 2 Diabetes Subtypes
Source: Int J Mol Sci. 2025 Jan 14;26(2):637. doi: 10.3390/ijms26020637 (PMC11766090; doi:10.3390/ijms26020637)
Supplement: Supplementary file 1 [file ijms-26-00637-s001.zip › ijms-3357245-supplementary figures.docx]

**Characterizing Circulating microRNA Signatures of Type 2 Diabetes subtypes**Fatima Sulaiman, Costerwell Khyriem, Stafny Dsouza, Fatima Abdul, Omer Alkhnbashi, Hanan Faraji, Muhammed Farooqi, Fatheya Al Awadi, Mohammed Hassanein, Fayha Salah Ahmed, Mouza Alsharhan, Abdel Rahman Tawfik, Amar Khamis and Riad Bayoumi^*^

**Supplementary Figures:**


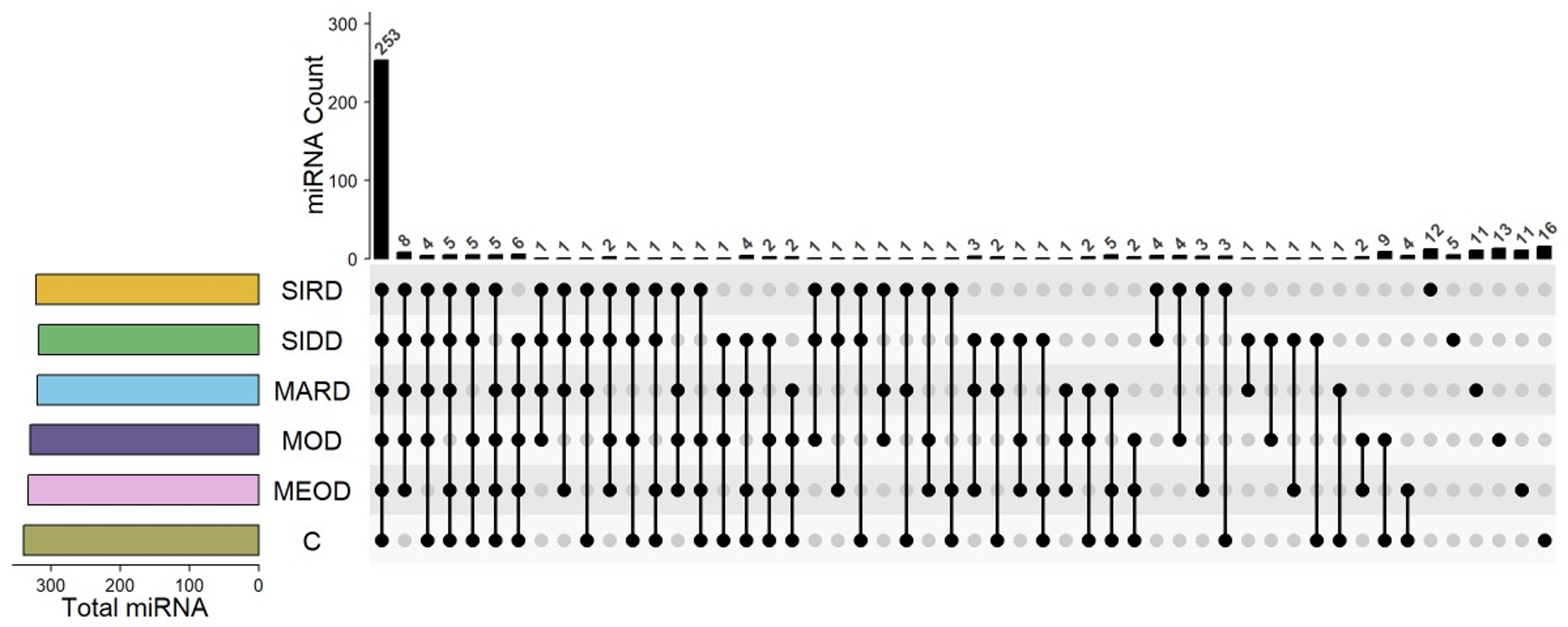


**Figure S1. Distribution of miRNAs identified in 45 T2D patients belonging to five T2D clusters and/or 7 Control samples.** The upset plot shows the numbers of miRNAs identified (n=430) along the Y-axis. A single black dot represents the number of miRNAs identified only in an individual cluster. Connected black dots represent shared miRNAs between clusters. Grey dots indicate that those miRNAs are not identified in that cluster. The clusters are labelled as Mild Early-Onset Diabetes (MEOD), Mild Age-Related Diabetes (MARD), Mild Obesity-related Diabetes (MOD), Severe Insulin-Resistant Diabetes (SIRD), Severe Insulin-Deficient Diabetes (SIDD) and Control (C).

*
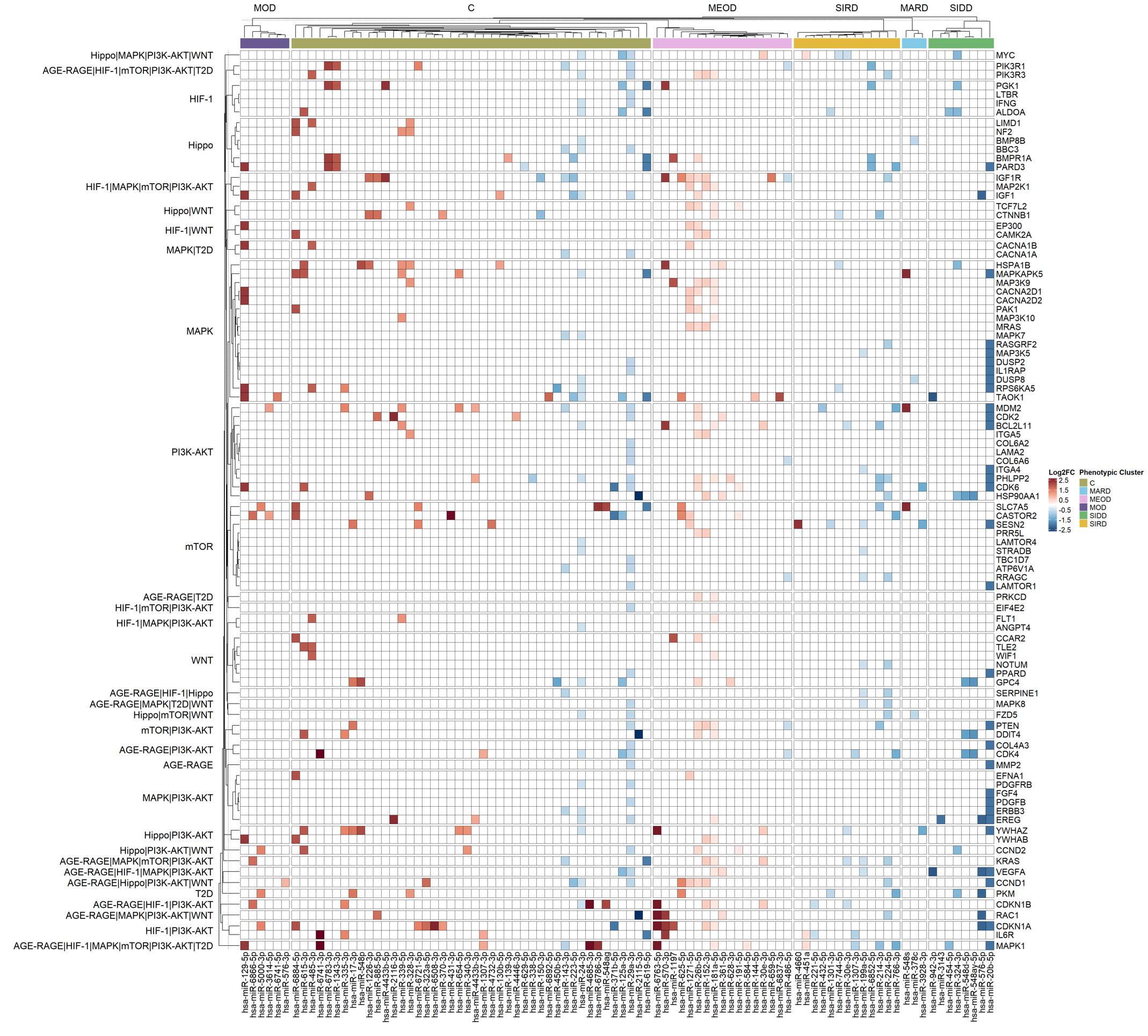
*

**Figure S2:** **Mapping of miRNA genes to target genes belonging to Type 2 diabetes related pathways**. Red color indicates up-regulation and blue indicates down-regulation of displayed miRNAs in the respective cluster.


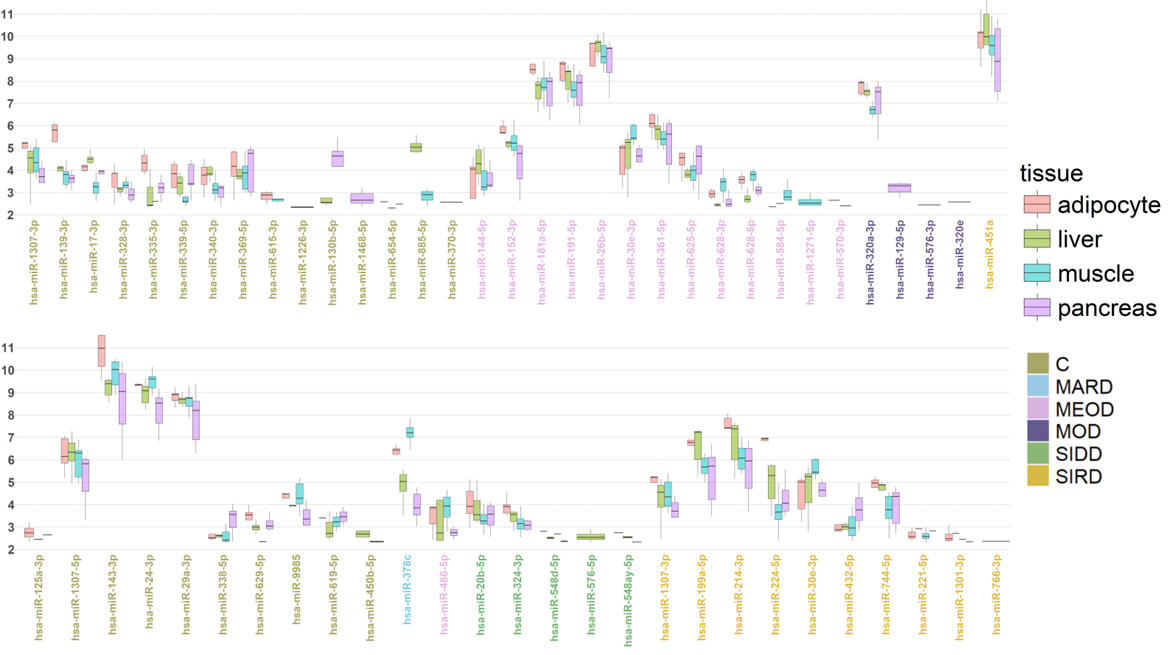


**Figure S3: Tissue-Atlas expression of differentially expressed miRNAs.** The upper panel shows upregulated miRNA tissue expression of inter-phenotypic T2D clusters. The lower panel shows down-regulated miRNAs amongst T2D clusters.

**A)**
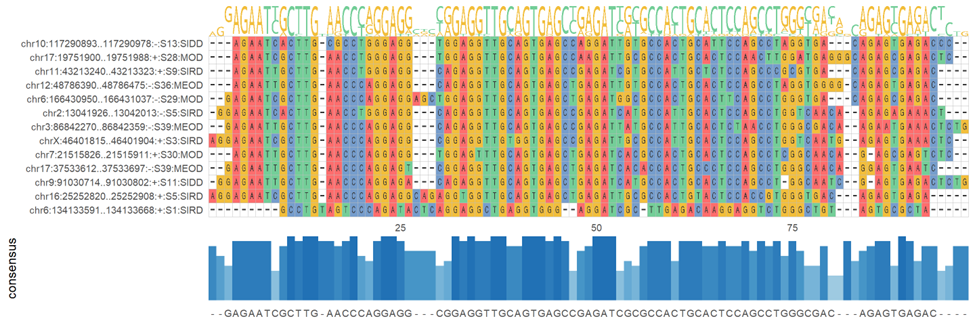


**B)**


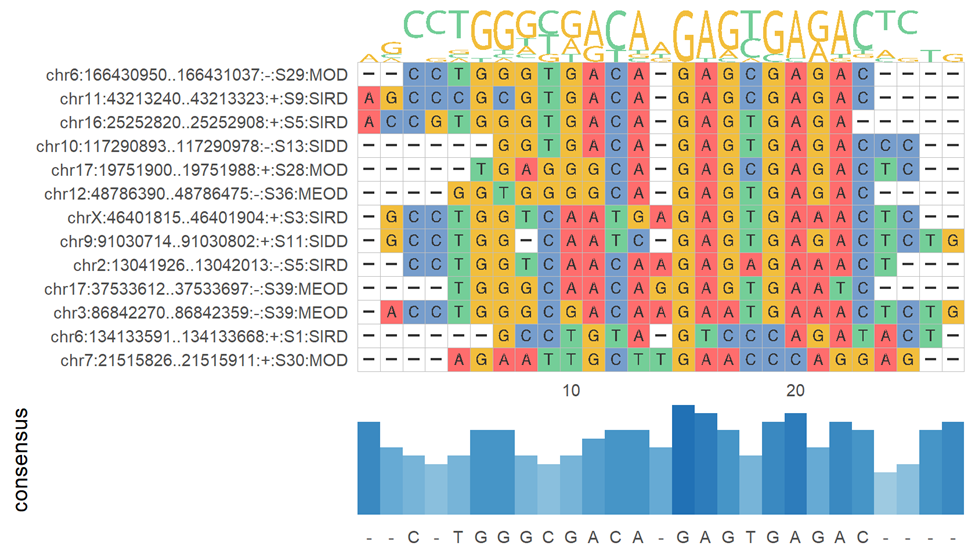


**Figure S4: Multiple sequence alignment of predicted novel miRNAs.** Multiple Sequence Alignment of 13 significant novel miRNAs A) Pre-Mature and B) Star Sequence is shown.
